# Study on water resources carrying capacity in Zhuanglang River Basin

**DOI:** 10.1007/s10661-022-10027-6

**Published:** 2022-04-22

**Authors:** Wen Xu, Chang Zhou, Bingrui Liu, Dongxue Wang, Xingzhu Zhao, Xiaojing Yang, Xiaotao Zhu, Zimu Lin

**Affiliations:** 1 Survey Bureau of Hydrology and Water Resources of Gansu Province, Lanzhou, 730000 China; 2 School of Accounting, Zhejiang University of Finance and Economics, Hangzhou, 310018 China; 3 College of Earth and Environmental Sciences, Lanzhou University, Lanzhou, 730000 People’s Republic of China; 4 Upstream Hydrology and Water Resources Bureau of Yellow River Conservancy Commission, Lanzhou, 730000 China; 5 School of Accounting, Anhui University of Finance and Economics, Bengbu, 233030 China; 6 Water Resources Department of Gansu Province, Lanzhou, 730000 China; 7 Hydrology and Water Resources Bureau of Gansu Province, Lanzhou, 730000 China

**Keywords:** Basin water resources, Carrying capacity, Quantitative calculation, Comprehensive evaluation

## Abstract

With the rapid development of China’s social economy, the phenomenon of unbalanced regional economic development is increasingly obvious. The shortage of water resources in northwest China is an important constraint to local development. The study on the water resources carrying capacity of the Zhuanglang River Basin plays an important role in the development of local economy; thus, we evaluate the water resources situation of Zhuanglang River Basin by using hydrology-related calculation method, combining with field investigation, visiting relevant departments, and referring to relevant data. Meanwhile, the water resources carrying capacity of 3 counties and 12 townships in the Zhuanglang River Basin in the current year are studied and analyzed based on methods such as fuzzy comprehensive evaluation model and quantitative calculation. Results indicate that the carrying capacity of water resources in Zhuanglang river basin can mostly meet the needs of township, but it is necessary to further optimize the water resources carrying capacity of the Zhuanglang River Basin from the four systems of water resources, society, economy, and ecology in the future. Besides, the results of quantitative calculation show that the maximum economic scale that water resources can withstand in most areas of the Zhuanglang River Basin is the overall well-off level, that is, the per capita domestic water is 80L/(d· person), and the per capita GDP is 10000 yuan/person. But there is still a certain gap to achieve the level, when the people are well-off in an all-round way, the two indexes are correspondently raised to 120L/(d· person), and the per capita GDP is 30,000 yuan/person.

**Supplementary information:**

The online version contains supplementary material available at 10.1007/s10661-022-10027-6.

## Introduction

With the rapid development of China’s economy, the unbalanced trend of regional economic development is increasingly obvious (Kuylenstierna et al., 1997). For example, the water demand continues to increase in Northwest China, where the economic and industrial structure is still dominated by the primary and secondary industries which consume more water. Therefore, the shortage of water resources has become an important restriction factor for the development of economic and social of this region. In order to solve the crisis of water resources and ensure the smooth progress of national economic construction, it is urgent to rationally evaluate the social and economic scale that water resources can bear (Qiao et al., 2021; Wang & Liu, 2019; Yang et al., 2019).

Water resources carrying capacity has a crucial influence on the comprehensive development and development scale of a country or region. Especially for the early dry and semi-arid areas with water shortage, it has become the “bottleneck” restricting its development (Bing et al., 2016; Guo, 2018; Nan, 2011; Wang, 2021). Therefore, the analysis, calculation, and evaluation of water resources carrying capacity become an important basis for seeking regional sustainable development (Mikhail, 2016; Su, 2017).

**Fig. 1:**
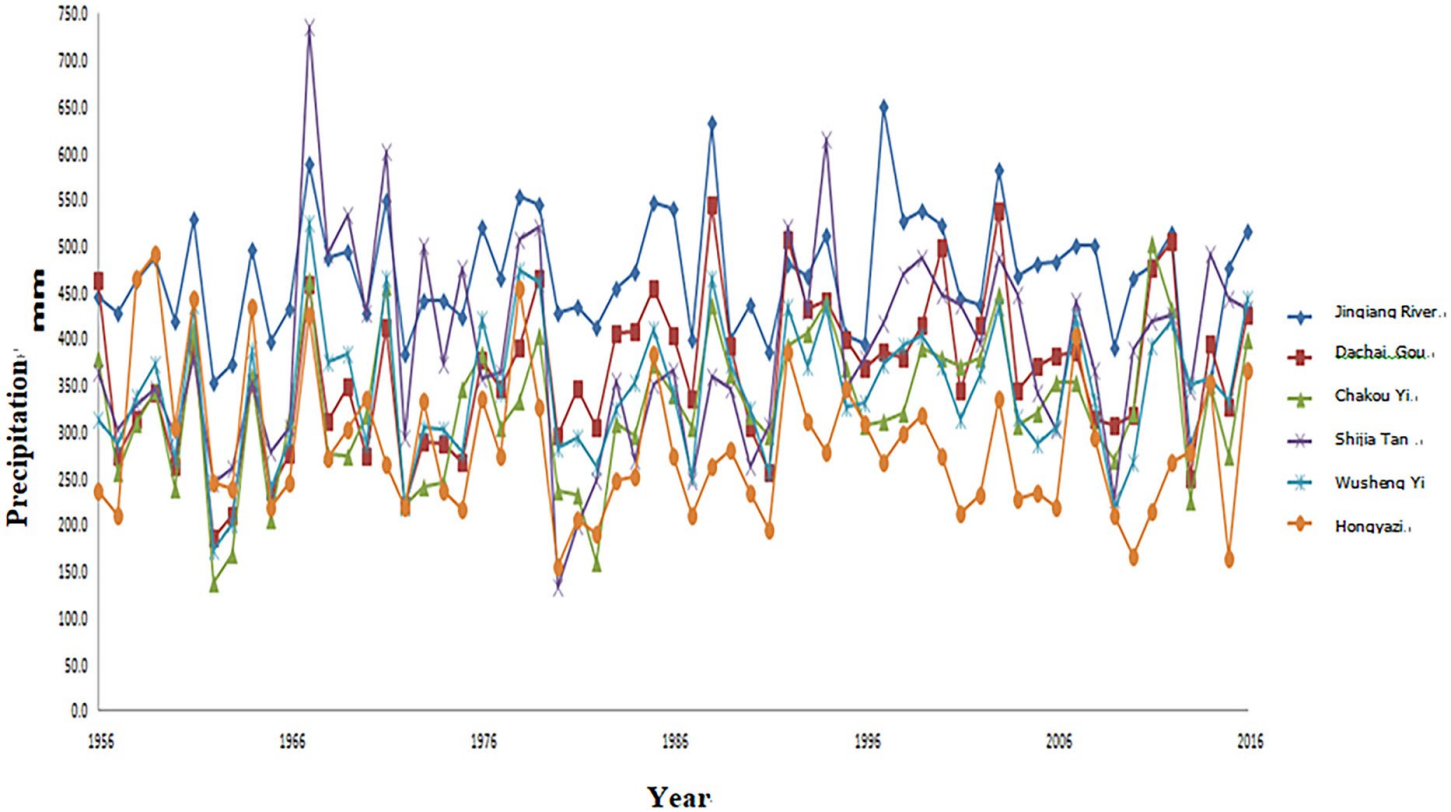
Annual precipitation process diagram of each station

**Fig. 2:**
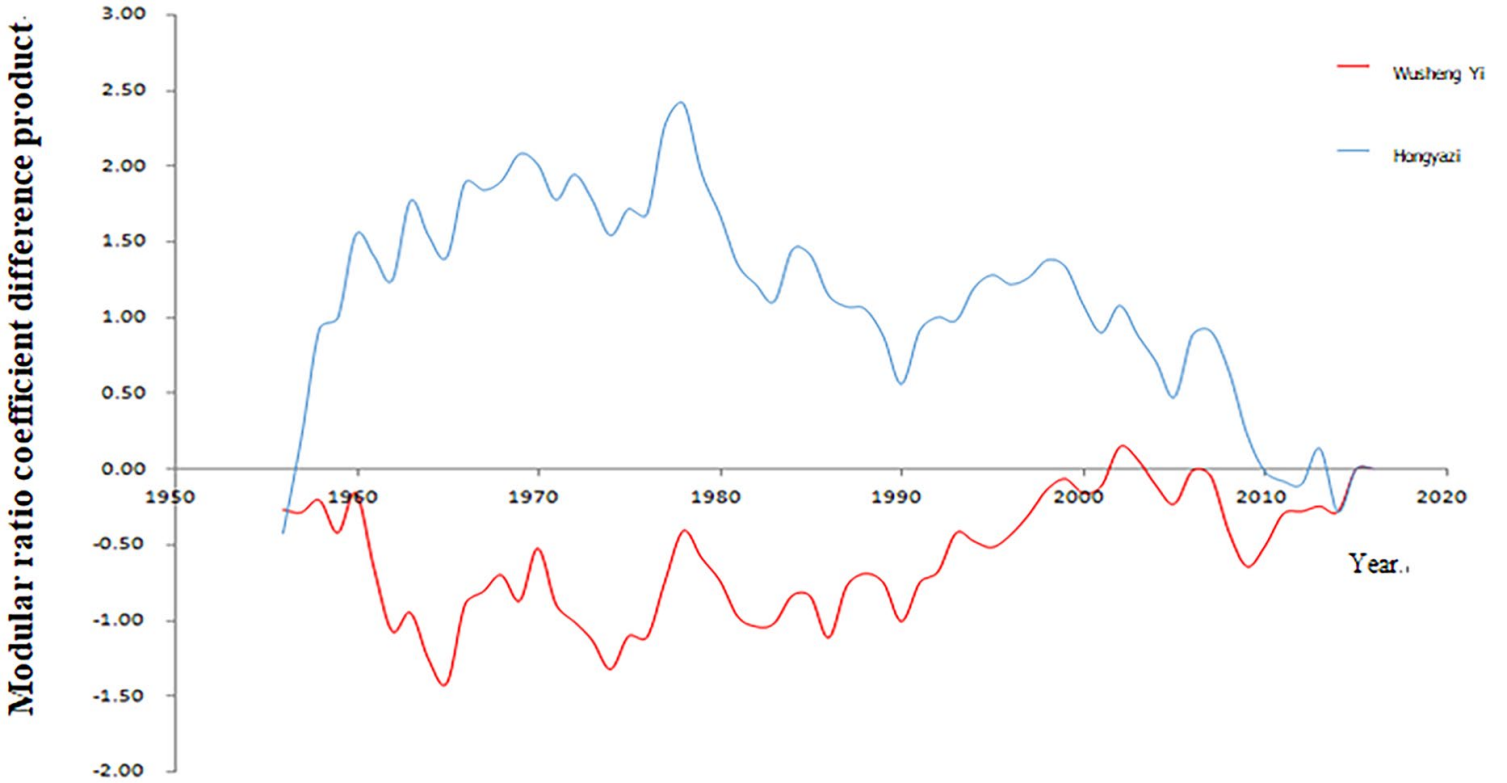
Represents the process diagram of the difference product of the station modulus ratio coefficient

As an economically developed area in Gansu Province and an important channel of the ancient Silk Road, the water resources in the Zhuanglang River Basin play a very important role in regional economic development and the implementation of the strategy of western development. Zhuanglang River Basin is located in the hinterland of Eurasia and has a continental climate. Meteorological factors are closely related to elevation changes. Precipitation increases with elevation, while evaporation and temperature decrease or decrease with elevation. Upper tianzhu Tibetan Autonomous County, the terrain is complex, the landscape is changeable, the climate has a strong vertical zonation. The high mountain area above 3000 m has an alpine and humid climate with abundant rainfall and little evaporation. The shallow valleys below 3000 m have a cold semi-arid climate with less rainfall and greater evaporation. According to the statistical analysis of the measured data of The Wuxuanling meteorological station (altitude 3045 m) for many years, the annual average temperature is -0.2 ℃, the annual extreme maximum temperature is 26.7 ℃, the extreme minimum temperature is -30.6 ℃, the annual average precipitation is 411.3 mm, evaporation is 1590.6 mm, and the average sunshine duration is 2571.3 h. Over the years, the maximum snow depth is 24 cm, the frozen soil depth is 149 cm, the average wind speed is 4.6 m/s, the maximum wind speed is 29.0 m/s, the frost-free period is 73d. The main climatic characteristics of this region are low temperature, short sunshine, short frost-free period, large temperature difference between day and day, and changeable weather. There are rainstorm, snowstorm, rime, rime and other phenomena, and natural disasters are more frequent. Most of yongdeng county in the middle and lower reaches belongs to temperate arid climate, while some areas in the northwest belong to alpine and semi-arid climate, with obvious climate differences. Majiping meteorological Station is represented by the annual average temperature of 5.9 ℃, maximum temperature of 13.5 ℃, minimum temperature of 0.0 ℃, annual average precipitation of 290.2 mm, evaporation of 1879.8 mm, average sunshine duration of 2659.3 h, annual average wind speed of 2.3 m/s, maximum wind speed of 20 m/s, frost-free period of 126d. The precipitation increases from southeast to northwest, and the temperature decreases from southeast to northwest. The illumination is longer than the full sunshine, the heat is not rich in temperature difference, the precipitation is scarce and the variability is large, and the continental climate disasters are the main climatic characteristics of this region. The precipitation in zhuanglang River Basin in recent years is shown in Figs. 1 and 2.

**Fig. 3:**
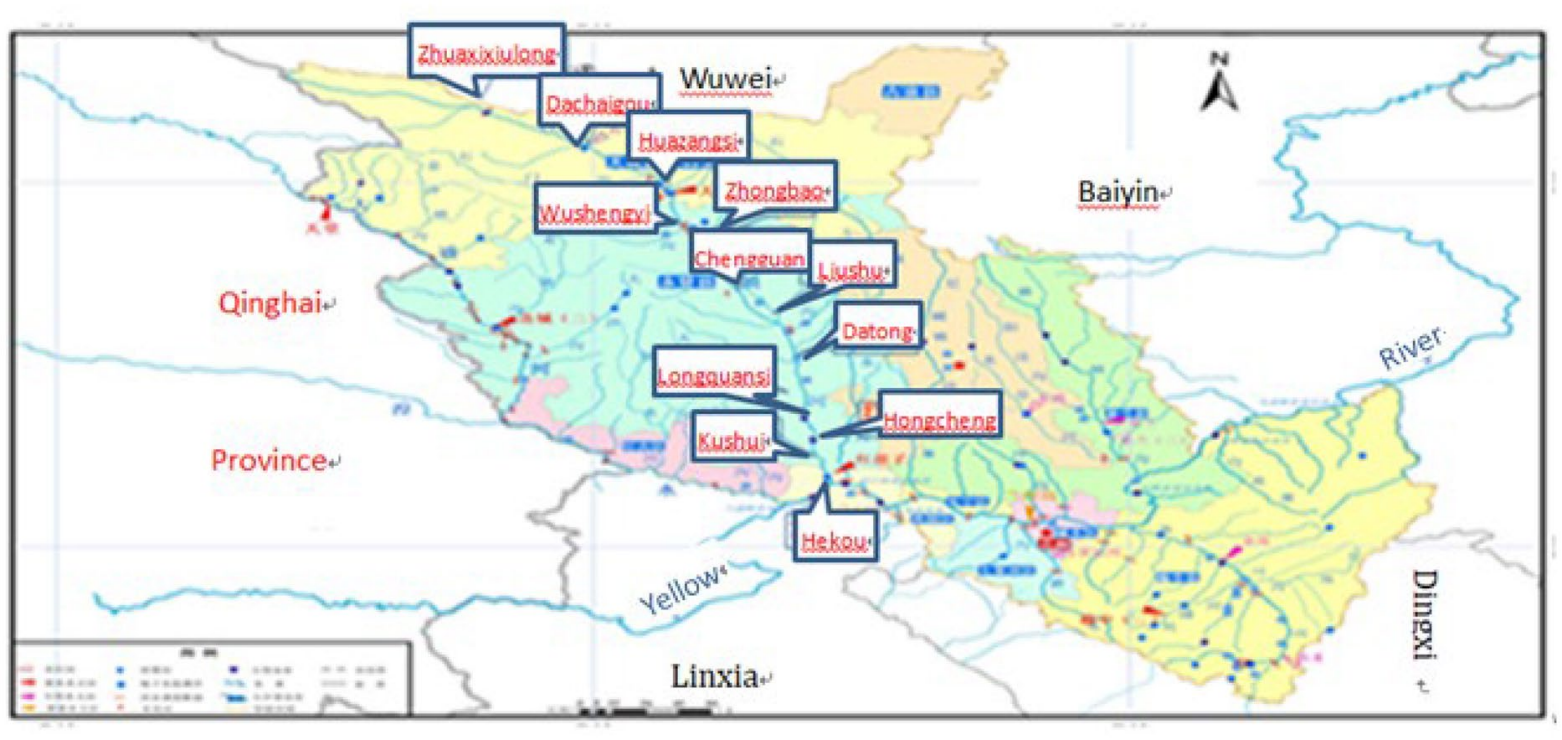
Geographic location of towns in Zhuanglang River Basin

**Fig. 4:**
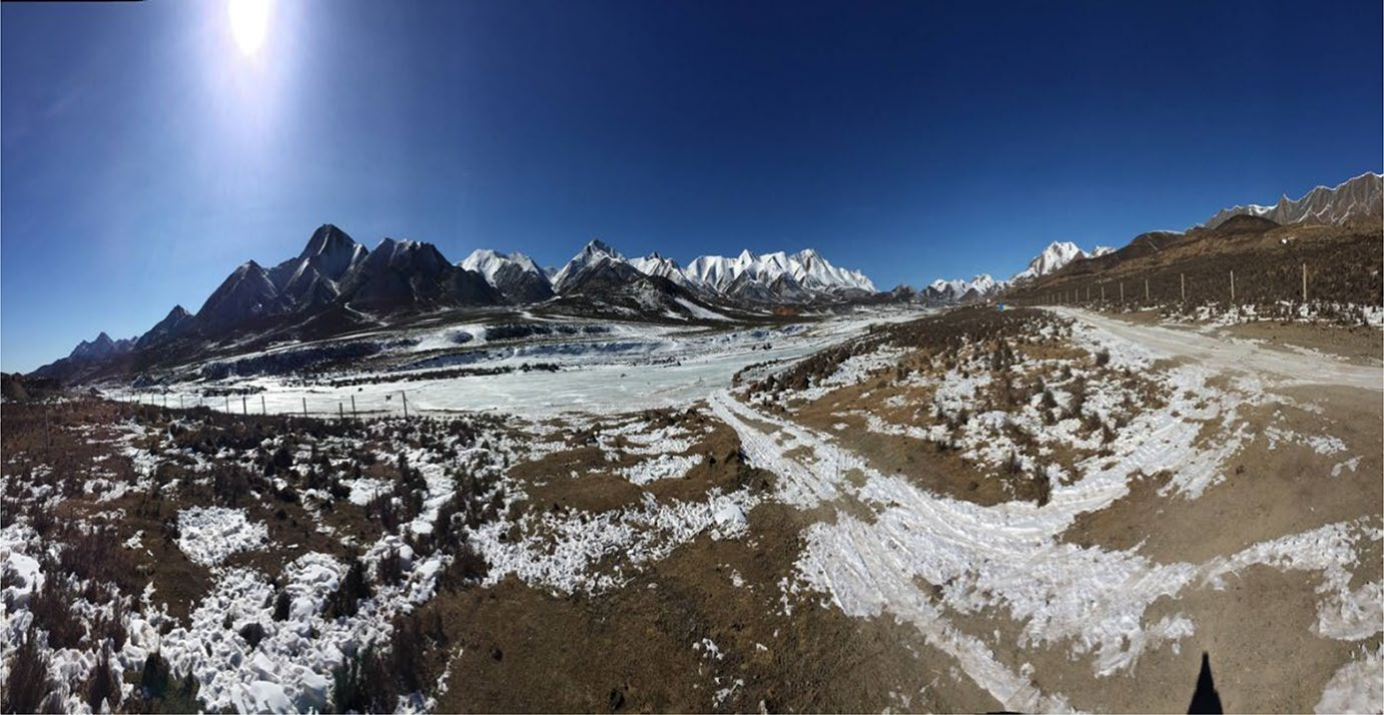
Scenic map of the source area of Zhuanglang River Basin

In this study, we explore the water resources carrying capacity and water ecological utilization value of Zhuanglang River combining with the social and economic indicators of each township in the basin by using the fuzzy comprehensive evaluation model (Ma, 2014; Yin, 2019) based on membership degree and calculating the water resources carrying capacity quantitatively (Han & Zhang, 2013). These results provide a scientific basis for water resources utilization, water ecological protection and water environment assessment in Zhuanglang River Basin (Liu et al., 2019; Zhao, 2010). The specific social and economic indicators of each town in the Zhuanglang River Basin are shown in Table S1. The geographical location of each township and the scenic map of the source area of Zhuanglang River Basin are shown in Figs. 3 and 4, respectively. And the amount of surface water and groundwater resources in each administrative area and their available conditions are shown in Tables S2 to S6, respectively. It is worth noting that the data level year is 2016, and all data are mainly from the local water conservancy department and statistics department. All the basic data are desensitized.

## Research methods, comprehensive evaluation, and quantitative calculation of water resources carrying capacity

### Methods

There are currently many research and analysis methods for water resources carrying capacity, and they are divided into two categories mainly: comprehensive evaluation and quantitative analysis (Bai, 2010; Chen et al., 2000; Huang et al., 2012; Sun, 2005; Yi et al., 2018). Comprehensive evaluation method is the key factor in the region were selected as the evaluation criteria, setting different values at the same time, will assess the numerical comparison and analysis with index, analysis of the various factors influencing the water resources carrying capacity size, and the membership grade of the standard is confirmed, the various standard level whole evaluation choice finally agreed on the level of the water holding capacity. It can more accurately obtain the level of water resources carrying capacity in the basin and the pros and cons of water resources carrying capacity in different regions. Quantitative calculation methods mainly take the maximum population and economic indicators carried by water resources as the main targets, and design the utilization level of various resources and local economic development level, so as to analyze and calculate the size of water resources carrying capacity. But it has the disadvantage of not being able to combine the actual reaction bearing capacity (Wu et al., 2020). According to the actual situation of the Zhuanglang River Basin, we analyze the water bearing capacity of the case area by applying related evaluation methods and quantitative calculations combined with the degree of attachment (Rijsberman & van de Ven, 2000).

The grading standard of most indicators in this study is determined by consulting the national standards or existing research results (Gao, 2015), and then adjusting some indicators according to the actual situation of the Zhuanglang River Basin, such as economic development, water resources quantity, and its development and utilization et.al (Zhiming et al., 2018). Based on the principle of water resources carrying capacity index, six standard levels have been identified. They are water resources system, social system, economic system (Zhiming et al., 2018), ecological environment system (Wang et al., 2019) and comprehensive coordination, respectively (Yang et al., 2020). And a total of 20 indicators from water resources system to comprehensive coordination have been established (Qiao, 2015), as is shown in Table 1.

**Table 1:** Evaluation standard index systems

Target layer	Criterion layer	Index layer	Unit	The meaning of selection
Water resources carrying capacity	Water Resources Subsystem	Water resource modulus	10000m^3^/km^2^	Basin water potential
		Utilization ratio of water resource	%	Status of Water Resources Examination and Utilization
		Water supply module	10,000 m^3^/km^2^	Basin water supply capacity
	Social subsystem	Population density	population/ km^2^	Reflect regional population pressure
		Urbanization rate	%	Social development level and population quality
		Population growth rate	%	Reflect the future population development trend
		Urban domestic water quota	L/ (person·d)	Urban population water consumption level
		Rural domestic water quota	L/ (person·d)	Water consumption level of rural population
	Economic subsystem	GDP per capita	10000yuan/ person	Reflect economic development
		Water consumption per 10,000 yuan industrial output value	m^3^/10000yuan	Reflect the level of industrial development
		Reuse rate of industrial water	%	Industrial development level and industrial water saving level
		Irrigation rate of plough	%	Development of regional farmland irrigation
		Grain yield per hectare	t/mu	Agricultural development level
		Water consumption per unit grain	m^3^/t	Utilization level of agricultural water resources
		Irrigation water quota	10000m^3^/mu	Development potential of agricultural water
	Ecological Environment Subsystem	Forest and animal husbandry coverage	%	The important index of regional environment reflects the renewal ability of water resources
		Groundwater extraction rate	%	Reflect the exploitation and utilization of groundwater
	Comprehensive coordination index	Water resources per person	m^3^/person	Abundant and deficient situation and development potential of water resources
		Water consumption per unit GDP	m^3^/10000yuan	The level of economic development and water resources utilization
		water consumption of ecosystem	%	Reflect ecological water usage

Data source: compiled by the author

The water resources carrying capacity of Northwest China is divided into five levels in this study. And the value range of each index under different levels and the carrying status of each level are shown in Table 2.

**Table 2:** Grading standard values of evaluation indexes

Target layer	Criterion layer	Index layer	Rank			
			1	2	3	4
Water resources carrying capacity	Water resources system	Water resource modulus	40	20	10	5
		Development and utilization rate of water resources	0.3	0.5	0.7	0.8
		Water supply modulus	25	15	10	5
	Social system	Population density	80	200	600	800
		Urbanization rate	0.7	0.5	0.3	0.1
		Population growth rate	0.2	0.4	0.7	1
		Urban domestic water quota	120	100	80	60
		Rural domestic water	90	60	40	20
	Economic system	GDP per capita	5000	10,000	25,000	40,000
		Industrial water consumption per 10,000 yuan of added value	10	30	70	100
		Reuse rate of industrial water	0.6	0.4	0.2	0.1
		Irrigation rate of plough	0.65	0.45	0.35	0.15
		Grain yield per hectare	0.3	0.2	0.1	0.05
		Water consumption per unit grain	1	1.5	2	2.5
		Irrigation water quota	200	300	400	500
	ecosystem	Forest and animal husbandry coverage	50	35	25	10
		Groundwater extraction rate	0.3	0.5	0.8	1
	Comprehensive coordination index	Water resources per capita	2000	1500	1000	500
		Water consumption per 10,000 yuan GDP	50	90	130	170
		Water consumption rate of ecology	0.15	0.1	0.05	0.03

Data source: compiled by the author

### Comprehensive evaluation

#### Fuzzy comprehensive evaluation method based on membership degree

The fuzzy comprehensive evaluation method was proposed based on the fuzzy mathematics (Qiao, 2015). In this method, on the basis of the membership theory of fuzzy mathematics, the indexes that are difficult to quantify in the comprehensive evaluation object, which can be converted into quantitative evaluation, and then according to different membership and weights and certain rules to carry on the overall evaluation (Ahmad et al., 2000). This method reflects all aspects of things, which avoids the information deviation and loss caused by a certain index evaluation, and systematically ensures the objectivity to the maximum extent.

Firstly, the principle and steps of this evaluation method are as follows:

1. There are n subareas in the evaluation basin. The expression of the sample set of subareas is D = {d1, d2, …, dn}, where dj (j = 1,2, n) represents the j-th partition.
2. There are m water resources carrying capacity indicators in each subregion:
  1 $$\mathrm{X}={\left({\mathrm{x}}_{\mathrm{ij}}\right)}_{\mathrm{m}\times \mathrm{n}}$$

where x_ij_ is the ith evaluation index value of j area, where i = 1, 2, 3, m; j = 1, 2, 3, …, n.

(3) According to the classification standard of water resources carrying capacity evaluation index (Yang & Yang, 2021), the index is divided into C level for identification, and then the identification standard of evaluation index can be expressed by matrix. The expression is as follows:where y_ih_ is the h-level critical value of the i-th index, h = 1, 2, c.
  2 $$\mathrm{Y}={\left({\mathrm{y}}_{\mathrm{ih}}\right)}_{\mathrm{m}\times \mathrm{c}}$$

(4) Setup construction of evaluation. The evaluation matrix Ri = (r_j_, k) _m × c_ is established to describe the membership degree of the j-th index in X corresponding to the k-th grade standard in Y, and then its value is derived according to the membership function. The membership functions are as follows:
  3 $${\mathrm{r}}_{1}=\begin{cases}{1}&{\mathrm{y}}_{1}\le \mathrm x\\ {\frac {\mathrm y_2{\mathrm {-x}}}{\mathrm y_2{\mathrm {-y_1}}}}& \mathrm y_2\le\mathrm x \le \mathrm y_1 \\ {0}& \mathrm x \le \mathrm {y_2}\end{cases}$$
  4 $${\mathrm{r_c}}=\begin{cases}{1}&{\mathrm{x}}\le \mathrm {y_c}\\ {\frac {\mathrm x{\mathrm {-y_{c-1}}}}{\mathrm {y_c}{\mathrm {-y_{c-1}}}}}& \mathrm {y_{c-1}}\le\mathrm x \le \mathrm {y_c} \\ {0}& \mathrm x \le \mathrm {y_{c-1}}\end{cases}$$
  5 $${\mathrm{r_h}}=\begin{cases}{\frac {\mathrm {y_{h-1}-\mathrm x}}{\mathrm {y_c}-\mathrm {y_{c-1}}}}&{\mathrm {y_{h-1}}}< \mathrm x < \mathrm {y_h}\\ {\frac {\mathrm x{\mathrm {-y_h}}}{\mathrm {y_{h+1}}-{\mathrm {y_h}}}}& \mathrm {y_h}<\mathrm x < \mathrm {y_{h+1}} \\ {0}& \mathrm x \le \mathrm {y_{h-1}}\;\mathrm {or}\;\mathrm {y_{h+1}}\le \mathrm x \end{cases}$$
(5) Calculate the index weight vector with binary mutual judgment matrix (Fang et al., 2019) and obtain the weighted vector by using entropy weight method (Cui et al., 2018):
  6 $${\mathrm{W}}_{1\times \mathrm{m}}={\omega }_{1},\;{\omega }_{2},\;\dots ,\;{\omega }_{\mathrm{m}}$$
(6) Fuzzy comprehensive evaluation: The fuzzy relation matrix Ri and the index weight matrix W are calculated compositely by fuzzy operator to obtain the comprehensive evaluation matrix Fn × C, which is composed of n C-dimensional row vectors Fi. Among them,
  7 $${\mathrm{F}}_{\mathrm{i}}={\mathrm{W}}_{1\times \mathrm{m}}\cdot {\mathrm{R}}_{\mathrm{m}\times \mathrm{c}}$$

Secondly, the index weight is determined by using the mutual judgment matrix.

1. The importance of different indicators at the same level is ranked qualitatively

The importance of different indicators hs and ht at the same level of the system is compared, and fst is used to express the qualitative ranking scale of importance. There are, if hs is more important than ht, fst = 1, fts = 0; if ht is more important than hs, fst = 0, fts = 1; if ht is as important than hs, fst = fts = 0.5, then fst + fts = 1, fss = ftt = 0.5. According to the comparison results, the importance order matrix of each index at the same level is established, as shown in Eq. (8).

8 $$\mathrm{F}=\left[\begin{array}{cccc}{\mathrm{f}}_{11}& {\mathrm{f}}_{12}& \cdots & {\mathrm{f}}_{1\mathrm{m}}\\ {\mathrm{f}}_{21}& {\mathrm{f}}_{22}& \cdots & {\mathrm{f}}_{2\mathrm{m}}\\ \vdots & \vdots & \ddots & \vdots \\ {\mathrm{f}}_{\mathrm{m}1}& {\mathrm{f}}_{\mathrm{m}2}& \cdots & {\mathrm{f}}_{\mathrm{mm}}\end{array}\right]$$

Meanwhile, meet the conditions:

9 $$\begin{cases}\mathrm{f_{hs}}>\mathrm{f_{ht}},\;\mathrm{f_{st}}=0\\\mathrm{f_{hs}}<\mathrm{f_{ht}},\;\mathrm{f_{st}}=1\\ \mathrm{f_{hs}}=\mathrm{f_{ht}}=0.5,\;\mathrm{f_{st}}=0.5 \end{cases}$$

The values of all the rows in the matrix F are summated, and then arranging the rows in the matrix f from large to small according to the sum value, so as to get the sorting under the condition of sorting consistency. In addition, for indicators of the same size, they represent the same importance and are sorted in order. The results are as follows: c’ = (c’1, c’2,…,c’m), where the c-value subscript represents the importance of sorting.

(2) Index weight calculation based on mood operator

According to the sorting result c and the sum of the comparison results of each index, the quantitative calculation adopts the binary comparison method based on complementarity proposed by Chen Shouyu et al. The calculation formula is shown in formula (9).

10 $$\begin{aligned}\mathrm{W}&=\left({\omega }_{1},\;{\omega }_{2},\;\dots ,\;{\omega }_{\mathrm{m}}\right)\\&=\bigg[\frac{1-{\mathrm{g}}_{\mathrm{l}1}}{{\mathrm{g}}_{\mathrm{l}1}}/{\sum }_{\mathrm{i}=1}^{\mathrm{m}}\frac{1-{\mathrm{g}}_{\mathrm{l}1}}{{\mathrm{g}}_{\mathrm{l}1}},\frac{1-{\mathrm{g}}_{\mathrm{l}2}}{{\mathrm{g}}_{\mathrm{l}2}}\\&/{\sum }_{\mathrm{i}=1}^{\mathrm{m}}\frac{1-{\mathrm{g}}_{\mathrm{l}1}}{{\mathrm{g}}_{\mathrm{l}1}},\dots ,\frac{1-{\mathrm{g}}_{\mathrm{lm}}}{{\mathrm{g}}_{\mathrm{lm}}}/{\sum }_{\mathrm{i}=1}^{\mathrm{m}}\frac{1-{\mathrm{g}}_{\mathrm{l}1}}{{\mathrm{g}}_{\mathrm{l}1}}\bigg]\end{aligned}$$

where,$\sum\nolimits_{\mathrm i=1}^{\mathrm m}\omega_{\mathrm i}=1,\;\omega_{\mathrm j}=\frac{1-{\mathrm g}_{\mathrm{lj}}}{{\mathrm g}_{\mathrm{lj}}}/\sum\nolimits_{\mathrm i=1}^{\mathrm m}\frac{1-{\mathrm g}_{\mathrm{lj}}}{{\mathrm g}_{\mathrm{lj}}}$, 0.5 ≤ g_lj_ ≤ 1.0 (i = 1,2, …, m) g_lj_ is the quantitative scale of the importance of Index c'l to c'j, as is shown in Table S7.

Finally, the objective weight is determined by entropy weight method (Zhou et al., 2017).

1. Suppose that there are m evaluation objects and each evaluation object has n evaluation indexes. The judgment matrix is constructed:

11 $$\mathrm{R}={\left({\mathrm{x}}_{\mathrm{ij}}\right)}_{\mathrm{mn}}\mathrm{i}=\mathrm{1,2},\;\dots ,\;\mathrm{ nj}=\mathrm{1,2},\;\dots ,\;\mathrm{m}$$

(2) The judgment matrix is normalized to get the normalized matrix, and its elements are as follows:

12 $${\mathrm{b}}_{\mathrm{ij}}=\frac{{\mathrm{x}}_{\mathrm{ij}}-{\mathrm{x}}_{\mathrm{imax}}}{{\mathrm{x}}_{\mathrm{imax}}-{\mathrm{x}}_{\mathrm{imin}}}$$

(3) According to the definition of information entropy, if there are m evaluation objects and n evaluation indexes, the i-th index (I = 1,2 n) (x_i1_, x_i2_) will be a comprehensive evaluation index in Xim, there are m states, and the information entropy corresponding to the i-th index is:
  13 $${\mathrm{H}}_{\mathrm{i}}=-\frac{\sum_{\mathrm{j}=1}^{\mathrm{m}}{\mathrm{f}}_{\mathrm{ij}}{\mathrm{lnf}}_{\mathrm{ij}}}{\mathrm{lnm}}, \mathrm{i}=\mathrm{1,2},\;\dots ,\;\mathrm{j}=\mathrm{1,2},\;\dots ,\;\mathrm{m}$$
  where $${\mathrm f}_{\mathrm i\mathrm j}=\frac{1+{\mathrm b}_{\mathrm i\mathrm j}}{\sum_{\mathrm j=1}^{\mathrm m}1+{\mathrm b}_{\mathrm{ij}}}$$.

(4) Calculate entropy weight of evaluation index

14 $${\omega }_{\mathrm{i}}=\frac{1+{\mathrm{H}}_{\mathrm{i}}}{\mathrm{n}-\sum_{\mathrm{i}=1}^{\mathrm{n}}{\mathrm{H}}_{\mathrm{i}}} ,\;\mathrm{ and\; meet \;the\; condition},\; \sum_{\mathrm{i}=1}^{\mathrm{m}}{\omega }_{\mathrm{i}}=1$$

#### Ability evaluation

According to the established evaluation system of water resources carrying capacity (Zhu et al., 2019), the water resources carrying capacity of 12 towns in Zhuanglang River Basin were evaluated by applying the fuzzy comprehensive evaluation method based on membership degree.

The 20 evaluation index values and standard values of 4 levels of these 12 towns are shown in Table 2.

### Quantitative calculation

It is necessary to carry out comprehensive analysis of water resources carrying capacity and quantitative calculation, due to comprehensive evaluation can only provide a qualitative result (Zhu et al., 2019). Based on the water resources situation, water demand of various industries and socio-economic data of 12 towns in Zhuanglang River Basin in 2016, we adopt the multi-objective water resources carrying capacity calculation model in this part (Zhang, 2019). The model evaluates the water resources carrying capacity of different living standards in that year with taking the population and economic scale of the basin as the measurement of water resources carrying capacity. Therefore, it provides the basis for the sustainable economic development planning of villages and towns in Zhuanglang River Basin.

#### Calculation methods

The calculation method used in this study is a multi-objective water resources carrying capacity calculation model (Wang et al., 2018), and the influence of the following factors is mainly calculated (Zhang et al., 2018). For example, we can judge the specific development and utilization status of water resources with the specific occurrence information of water resource in the basin. Moreover, the specific structure and the related efficiency coefficient of water can be defined to clarify the specific water demand. The actual occurrence state of water resources in a watershed will be affected by the specific amount of available water resources. The development coefficient and the corresponding water diversion situation can be expressed by the following formula:

15 $${W}_{t}={W}_{o}+{W}_{s}\cdot {\alpha }_{s}+{W}_{g}\cdot {\alpha }_{g}$$

where W_t_ is the total amount of regional available water resources; W_s_ and W_g_ represent the total amount of surface water and surface water resources in a certain level year, respectively; α_s_ and α_g_ are the exploitation and utilization coefficients of surface water and groundwater respectively, in addition 0 ≤ α_s_ and α_g_ ≤ 1; and W_o_ is the amount of water transferred out of the region.

The structure of regional water refers to the distribution of water resources in three industries, domestic water and environmental water. The distribution coefficient matrix of water consumption is as follows:

16 $${\beta }_{t}^{k}=( {\beta }_{ti}^{k}{\beta }_{ta}^{k}{\beta }_{ts}^{k}{\beta }_{tl}^{k}{\beta }_{te}^{k})$$

where ${\upbeta }_{\mathrm{t}}^{\mathrm{k}}$ represents the k-th water distribution scheme of a certain level year in the region; β_ti_, β_ta_, β_ts_, β_tl_, β_te_ are the water distribution coefficients of industrial, agricultural, tertiary, domestic and environmental water distribution schemes, respectively; and 0 ≤ β_ti_, β_ta_, β_ts_, β_tl_, β_te_ ≤ 1,the sum of them is 1.

Water efficiency coefficient represents the water resource utilization rate and efficiency of each water consumption object. It is expressed by matrix Uwt:

17 $${U}_{wt}=({U}_{ti}{U}_{ta}{U}_{ts}{U}_{ tl}{U}_{te})$$

where the efficiency coefficient of water of industry, agriculture and the third industry and the output value per cubic meter are expressed as U_ti_, U_ta_, U_ts_, U_tl_ and U_te_ respectively, and the unit is yuan / m^3^; the efficiency coefficient of domestic water, U_tl_, is the reciprocal of the per capita domestic water quota, and the unit is person/ m^3^; the benefits of environmental water use are comprehensive benefits, which have been included in industry, agriculture and the tertiary industry, therefor U_te_ = 0, the value can avoid repeated calculation.

The supporting capacity of regional water resources to water users under the k-th water distribution scheme can be expressed as:

18 $${Z}_{Wt}^{k}=({Z}_{ti}^{k}{Z}_{ta}^{k}{Z}_{ts}^{k}{Z}_{tl}^{k}{Z}_{te}^{k})$$

where ${\mathrm{Z}}_{\mathrm{Wt}}^{\mathrm{k}}$=Wt⋅${\upbeta }_{\mathrm{t}}^{\mathrm{k}}$⋅ (Uwt)_T_, (Uwt)_T_ is the transpose of Uwt.

Generally speaking, the regional water resource availability (Wt) and water efficiency coefficient (Uwt) are relatively constant in a certain level year (Tian et al., 2021). Different water distribution schemes will determine the capacity of regional water resources. According to the definition of water resources carrying capacity, the maximum supporting capacity is max ( kWtZ) that is water resources carrying capacity (Wu, 2017).

It is necessary to unify each component under a unified standard due to the incommensurability of water resources to each water object in different carrying capacity units. Generally, the water resources carrying capacity of each water object needs to be converted into the number of people (Cao et al., 2020). Therefore, a vector R_T_ is constructed, which includes the per capita demand of all aspects of society by introducing the per capita demand vector.

19 $${R}_{t}=({R}_{ti}{R}_{ta}{R}_{ts}{R}_{tl}{R}_{te})$$

where R_tj_ the per capita demand of a water object in a certain level year; R_ti_, R_ta_, R_ts_, R_tl_, R_te_ represent the per capita demand of industrial, agricultural, tertiary, domestic and environmental water respectively; Rti, Rta, and Rts are the per capita output value of agriculture, industry and tertiary industry respectively, and the unit is yuan / person; R_tl_ is the product of per capita domestic water demand and domestic water efficiency coefficient, unit: 1; R_te_ is the same as R_tl_, unit: 1.

The population that water can carry is a comprehensive index to evaluate its carrying capacity (Cao et al., 2020). Regional water resources carrying capacity is the largest population that water resources can support under different water distribution schemes. Therefore, the calculation model is as follows:

$$\mathrm{OB}.{P}_{t}=\underset{k}{\mathrm{max}}\left(\frac{{Z}_{{W}_{tj}}^{k}}{{R}_{tj}}\right)$$

20 $$\mathrm{S}.\mathrm{T}. \sum {\beta }_{{W}_{tj}}^{k}\le 1.0$$

$$0\le {\beta }_{tj}^{k}\le 1.0$$

where P_mt_ refers to the maximum population of regional water resources in a certain level year, j = I、a、s、l、etheir values represent the five water consumption objects of industry, agriculture, tertiary industry, domestic water and environmental water respectively. Considering the priority of environmental water, the model is simplified and solved by substituting relevant parameters:

21 $${\mathrm{P}}_{\mathrm{mt}}=({W}_{t}-{W}_{te})/\sum \frac{{R}_{tj}}{{U}_{{W}_{tj}}}$$

where W_t_ refers to the total amount of water resources available in a region in a year, while W_te_ refers to the environmental water consumption in a certain level year.

The above model can analyze and obtain the specific water resources carrying level in the evaluation area. And the actual carrying information can be expressed by the corresponding relative carrying index (RCI) (Liao et al., 2020). It represents the magnitude of the current or subsequent specific stage and the predicted bearing capacity value. The actual analytical formula is:

22 $${\mathrm{RCI}}_{\mathrm{t}}=\frac{{\mathrm{CCP}}_{\mathrm{t}}}{{\mathrm{P}}_{\mathrm{mt}}}$$

where RCI_t_ is the relative carrying index of water resources in a certain level year and CCP_t_ is the total population in a certain level year. The specific parameters of reference RCI value can be divided into three basic types: (1) When RCI > 1, the actual carrying capacity of water resources is lower than the specific parameters, it is overload; (2) When RCI = 1, the bearing capacity value is equal to the corresponding value, which forms a special balance, namely the critical value; (3) When RCI < 1, that is, the bearing capacity exceeds the actual parameters, namely the lack of load. Overload and lack of load can be further divided into three types: strong, medium and weak, the detailed standards are shown in Table S9.

#### Calculation of water resources carrying capacity of Zhuanglang River Basin in current year

The water resource efficiency coefficient of each industry was calculated by using formula (17) based on the water demand and output value of each industry in Zhuanglang River Basin in 2016. And the proportion of industry, agriculture and third industries in the national economy were also calculated, as shown in Tables 6 and 7.

## Results and discussion

The weight of five indexes in criterion layer is determined by binary mutual judgment matrix, and then the binary mutual judgment matrix of criterion layer is obtained by expert scoring method. We sum and sort the mutual judgment values, and calculate the weight of five indexes according to formula (10) based on columns 2–5 in Table 3. It can be seen from Table 3 that the weight of water resources subsystem is the largest in the criteria layer of water resources carrying capacity system, which is 0.32. Secondly, the second is the social subsystem and the economic subsystem, both of which are 0.18. Finally, the weight of the ecological subsystem and the comprehensive coordination index is the smallest, which is 0.16. It shows that water resources, as the resource provider of water resources carrying capacity, has the greatest impact on water resources carrying capacity. However, different utilization mode and structure of water resources of social and economic systems will also have a significant impact on water resources, while the impact of ecological environment and comprehensive coordination index is relatively small (Mou et al., 2020).

**Table 3:** Weight calculation table of each subsystem in the criterion layer

Criterion layer	B1	B2	B3	B4	B5	sum	sort	g_i_	(1-g_i_) /g_i_	ω_i_
B1	0.5	1	1	1	1	4.5	1	0.50	1.00	0.32
B2	0	0.5	0.5	1	1	3	2	0.65	0.54	0.18
B3	0	0.5	0.5	1	1	3	2	0.65	0.54	0.18
B4	0	0	0	0.5	0.5	1	3	0.68	0.47	0.16
B5	0	0	0	0.5	0.5	1	3	0.68	0.47	0.16

Data source: compiled by the author

According to the data of different indicators in each region in Table S8, the weight of each indicator obtained by entropy weight method are shown in column 6 of Table 4. It has been seen that most of the index weights are close, but some of them are quite different with comparing the subjective weight of binary mutual judgment method (Sun et al., 2020) with the objective weight of entropy method (Deng et al., 2020). The comprehensive weight is obtained by adding the subjective weight and objective weight according to the specific gravity of 0.5.

**Table 4:** Weight of each index

Subjective weight of binary mutual judgment method					Entropy	Comprehensive weight
Criterion layer	Criteria layer weight	Index layer	Index layer weight	Weight		
Water resources subsystemU_1_	0.256	U_11_	0.396	0.1264	0.0576	0.092
		U_12_	0.2136	0.068	0.0456	0.0568
		U_13_	0.1904	0.0608	0.0496	0.0552
Social subsystemU_2_	0.144	U_21_	0.2784	0.0504	0.0384	0.044
		U_22_	0.1496	0.0272	0.0472	0.0368
		U_23_	0.1192	0.0216	0.0296	0.0256
		U_24_	0.1192	0.0216	0.0216	0.0216
		U_25_	0.1336	0.024	0.0384	0.0312
Economic subsystemU_3_	0.144	U_31_	0.2008	0.036	0.0424	0.0392
		U_32_	0.108	0.0192	0.0272	0.0232
		U_33_	0.0944	0.0168	0.0432	0.0304
		U_34_	0.0864	0.0152	0.0376	0.0264
		U_35_	0.108	0.0192	0.0264	0.0232
		U_36_	0.0944	0.0168	0.0288	0.0232
		U_37_	0.108	0.0192	0.024	0.0216
Ecological environment subsystemU_4_	0.128	U_41_	0.52	0.0832	0.0096	0.0464
		U_42_	0.28	0.0448	0.0336	0.0392
Comprehensive coordinationU_5_	0.128	U_51_	0.396	0.0632	0.0456	0.0544
		U_52_	0.2136	0.0344	0.0456	0.04
		U_53_	0.1904	0.0304	0.0368	0.0336

Data source: compiled by the author

The water resources carrying capacity of 12 townships in Zhuanglang river basin is listed in Table 5 according to the principle of maximum membership degree. The results show that zhuaxixiulong Township and Dachaigou Town have higher water resources carrying capacity, belonging to the first level among the 12 townships in Zhuanglang River Basin. From this, it indicates that the structure of water resources utilization is reasonable and the development and utilization potential of water resources is huge. And then the water resources carrying capacity of huazangsi Town, wushengyi Town, Hongcheng Town and Hekou Town belongs to the second level, which indicates that the water resources situation in this area is relatively good. Finally, Zhongbao Town and Chengguan Town have the lowest level of water resources carrying capacity, which belongs to the fourth level, indicating that the county’s water resources are in a state of serious carrying capacity in the current year. Moreover, the rest of the townships belong to the third level, they are weak overload.

**Table 5:** Results of fuzzy comprehensive evaluation

County-level administrative region	Serial number	Townships	1	2	3	4	Maximum subordinate level
Tianzhu County	1	Zhuaxixiulong township	0.321	0.154	0.165	0.137	1
	2	Dachaigou town	0.213	0.188	0.205	0.156	1
	3	Huazangsi town	0.202	0.241	0.231	0.192	2
Yongdeng County	1	Wushengyi town	0.177	0.278	0.187	0.243	2
	2	Zhongbao town	0.126	0.254	0.226	0.268	4
	3	Chengguan town	0.249	0.308	0.174	0.331	4
	4	Liushu town	0.242	0.206	0.283	0.165	3
	5	Datong town	0.206	0.228	0.325	0.169	3
	6	Longquansi town	0.154	0.292	0.314	0.101	3
	7	Hongcheng town	0.156	0.355	0.323	0.125	2
	8	Kushuui town	0.326	0.188	0.361	0.122	3
Xigu District	1	Hekou town	0.102	0.378	0.295	0.285	2

Data source: compiled by the author

It can be seen from Tables 6 and 7 that agriculture, industry and the tertiary industry account for 20%, 40%, and 40% of the national economy respectively, from the perspective of the overall economic structure of Zhuanglang River Basin. Among them, industry and the tertiary industry account for a large proportion of the national economy. In addition, the economic development of Zhuaxixiulong Town and Dachaigou Town in the upper reaches of Zhuanglang River is dominated by agriculture, and accounting for 42% and 39% of the total output value respectively. Such as Zhongbao Town, Chengguan Town, Hongcheng Town, and Kushui Town, which locate in the middle and lower reaches, their agricultural output value accounts for less than 20% of the GDP. Meanwhile, their proportion of industrial output value is relatively high, especially in Zhongbao Town, reaching more than 85.39%. But the output value of the tertiary industry of these towns is generally more than 40%. Results indicate that the economic structure of these towns is that industry and the tertiary are relatively developed, while the proportion of agriculture is relatively low. The agricultural, industrial and the tertiary industry output value of Longxiang Town, Liushu Town, Datong Town, Longquansi Town and Hekou Town account for 30%, 20%, below and 50% respectively, indicating that the economic structure of these three counties is manifested as high proportion of agriculture and underdeveloped industry. Besides, the agricultural output value of Huazang Temple Town and Wushengyi Town account for between 20 and 30%. In a word, the characteristics of the economic structure of these areas are as follows. For example, the proportion of industry is similar, the industrial structure is not reasonable, and the economic level is underdeveloped.

**Table 6:** Water use situation of each township in Zhuanglang River Basin in current year

County- level administrative region	Serial number	Townships	Water availability (10000m^3^)	Water consumption (10000m^3^)				
				Agriculture	Industry	The tertiary industry	Domestic	Ecology
Tianzhu County	1	Zhuaxixiulong township	3141.407	564	28	0.08	3.312	12.152
	2	Dachaigou town	2105.527	499.2	167.328	0.32	12.56	27.44
	3	Huazangsi town	2285.444	716	227.8	3.44	30.32	39.76
Yongdeng County	1	Wushengyi town	1832.589	695.2	336.96	7.12	48	22.048
	2	Zhongbao town	215.2008	567.2	861.52	2.16	51.2	14.952
	3	Chengguan town	107.4546	320.8	128.64	25.6	142.16	12.072
	4	Liushu town	1108.08	1120.8	68.112	5.52	51.6	25.32
	5	Datong town	623.295	1328	102.384	4.72	50.48	30.224
	6	Longquansi town	379.2258	1219.2	24.64	2.48	46.4	13.032
	7	Hongcheng town	699.7817	834.4	60.72	5.12	50.48	19.296
	8	Kushuui town	582.5002	904	66.56	12	50.24	20.832
Xigu District	1	Hekou town	1.98288	16.8	0	0	16.8	3.392

Data source: compiled by the author

**Table 7:** Output value and efficiency coefficient of each township in Zhuanglang River Basin in current year

County-level administrative region	Serial number	Townships	Output value (100 million yuan)			Industry proportion			Efficiency coefficient (yuan/m^3^)		
			Agricult-ure	Industry	The tertiary industry	Agric-ulture	Industry	The tertiary industry	Agric-ulture	Industry	The tertiary industry
Tianzhu County	1	Zhuaxixiulong township	0.376	0.376	0.136	0.336	0.336	0.12	5.336	25.12	896
	2	Dachaigou town	0.576	0.376	0.536	0.312	0.2	0.288	9.232	17.92	1344
	3	Huazangsi town	1.056	0.904	2.672	0.184	0.16	0.464	11.8	31.84	2226.4
Yongdeng County	1	Wushengyi town	2.144	4.32	2.248	0.2	0.4	0.208	24.672	102.56	2524
	2	Zhongbao town	0.712	9.68	0.944	0.048	0.68	0.064	10.04	89.92	3496
	3	Chengguan town	0.568	0.96	4.944	0.072	0.12	0.608	14.168	59.68	2744.8
	4	Liushu town	1.296	0.688	1.776	0.272	0.144	0.376	9.248	80.8	2572.8
	5	Datong town	1.112	0.648	1.712	0.256	0.152	0.392	6.696	50.64	2907.2
	6	Longquansi town	1.16	0.32	1.464	0.312	0.088	0.4	7.608	103.92	2818.4
	7	Hongcheng town	0.68	0.88	2.136	0.144	0.192	0.464	6.52	115.92	3337.6
	8	Kushuui town	0.944	2.56	2.864	0.12	0.32	0.36	8.352	307.68	1906.4
Xigu District	1	Hekou town	0.008	0	0	0.76	0.04	0	3.808	0	0
		Synthesize	10.632	21.712	21.424	0.16	0.32	0.32	9.68	83.808	2476.8

Data source: compiled by the author

The efficiency coefficient of agricultural water use of Dachaigou, Huazangsi, Wushengyi, Zhongbao, Chengguan, Liushu and Kushui towns in Zhuanglang river basin is more than 10 yuan / m^3^, which is relatively high, according to the analysis of water efficiency coefficient. However, for the Zhuaxixiulong Township, Longquansi Town, Datong Town, Hekou Town and Hongcheng Town, the corresponding value of the coefficient is no more than 10 yuan / m^3^. The reason for this phenomenon is that the facilities in economically developed areas are more complete and the utilization rate of irrigation water is higher, while the source area and estuary area have sufficient irrigation water, which shows a problem about waste of water, and the efficiency coefficient is not high. The industrial development in Zhuaxixiulong Township, Dachaigou Town and Huazangsi is relatively backward, and the coefficient in the industrial field is obviously low, less than 50 yuan / m^3^, in terms of industrial water consumption. The efficiency coefficient of industrial water of Kushui Town, Wushengyi Town, Zhongbao Town, Hongcheng town and Longquansi Town is higher. On the other hand, the water efficiency coefficient of tertiary industry of the tertiary industry in the economically developed middle and lower reaches is generally more than 3000, while the water use efficiency coefficient of the two source towns is less than 2000, which is more backward than that of the middle and lower reaches.

According to the economic development model of the basin, it is initially set under the overall well-off baseline, that is, the per capita water consumption is 80L / (D · person), and the per capita GDP is 10000 yuan / person. If we are well-off in an all-round way, the two indicators are correspondingly raised to 120L / (D · person), and the per capita GDP is 30000 yuan / person. Based on the analysis and calculation of the per capita demand and the efficiency coefficient of different industries, the carrying population and current water resources carrying information of each region under different living standards are obtained according to formula (21). The detailed information is referred to Table 8.

**Table 8:** Water resources carrying capacity of towns in Zhuanglang River Basin under current situation, overall well-off level, and overall well-off level

County-level administrative region	Serial number	Townships	The actual populati-on	Current living standard		Overall well off		Well off in an all round way	
				Bearable population	RCI	Bearable population	RCI	Bearable population	RCI
Tianzhu County	1	Zhuaxixiulong township	0.336	7.192	0.04	4.144	0.064	1.352	0.2
	2	Dachaigou town	1.344	5.104	0.208	3.784	0.28	1.488	0.72
	3	Huazangsi town	5.344	7.52	0.568	4.048	1.056	1.672	2.552
Yongdeng County	1	Wushengyi town	2.808	3.68	0.608	2.408	0.936	0.944	2.376
	2	Zhongbao town	1.936	0.352	4.368	0.272	5.752	0.096	16.152
	3	Chengguan town	3.8	0.2	15.488	0.184	16.208	0.064	46.56
	4	Liushu town	2.216	1.568	1.128	1.096	1.616	0.408	4.352
	5	Datong town	2.144	1.064	1.608	1.256	1.368	0.464	3.704
	6	Longquansi town	1.832	0.768	1.912	1.784	0.824	0.696	2.104
	7	Hongcheng town	2.136	1.392	1.224	1.416	1.208	0.504	3.384
	8	Kushuui town	2.6	1.096	1.904	0.816	2.552	0.288	7.304
Xigu District	1	Hekou town	0.16	0.344	0.376	0.24	0.528	0.088	1.504

Data source: compiled by the author

Zhongbao, Chengguan, Liushu, and Kushui towns are at the level of strong overload, while Datong town and red town are at the middle and strong overload level when under the overall well-off living standard. It indicates that the specific situation of water resources carrying capacity in the areas is relatively severe. Therefore, it is necessary to pay full attention to the control of industrial structure and improve the overall water efficiency in the process of economic construction. Moreover, Zhuaxixiulong Township, Dachaigou Town and Hekou Town are at the level of load shortage, which indicates that there is a large space for water resources utilization and economic development in these towns in the current year. Under the overall well-off living standard, Huazangsi Town, Wushengyi Town, Zhongbao Town, Chengguan Town, Liushu Town, Datong Town, Longquansi Town, Hongcheng town and Kushui town are in a strong overload level. Hekou Town is of weak overload level, Dachaigou Town is of medium underload level, and Thuaxixiulong Town is of strong underload level. It can be seen that the water resources of townships cannot bear the overall well-off economy and population scale from the existing economic structure and water efficiency coefficient of various industries, comparing with the results of current living standards. So it is important that adjusting the economic structure, improving the efficiency coefficient of water use in various industries and the carrying capacity of water resources. Otherwise, it will face serious water shortage.

## Conclusions

The water resources carrying capacity of 12 towns in 3 counties of Zhuanglang River Basin were evaluated through the fuzzy comprehensive evaluation model based on membership degree. It is concluded that Zhuaxixiulong Township and Dachaigou Town have higher water resources carrying capacity and great potential for water resources development and utilization. And the water resources carrying capacity of Huazangsi Town, Wushengyi Town and Hekou Town belongs to the second level, and the water resources situation in this area are relatively good. However, Chengguan Town and Zhongbao Town have the lowest water resources carrying capacity, which is relatively poor in the current year. In addition, the rest of the towns are in the third level, which belongs to weak overload, and the overall water resources carrying capacity is relatively poor. Although the water demand in the middle and lower reaches of Zhuanglang river basin is relatively large, the water production in the upper reaches of Zhuanglang river basin is relatively high and the utilization efficiency of water resources is relatively low. This will cause the problem that the inflow volume of middle and lower reaches become rich, the available amount increase, and then the water resources carrying capacity is improved. In general, most of the water resources carrying capacity of Zhuanglang river basin can meet the water demand of villages and towns, but it is still necessary to further optimize the water resources carrying capacity of Zhuanglang River Basin from four aspects of water resources subsystem, social subsystem and ecosystem.

The results show that the largest economic scale that most areas of Zhuanglang river basin can bear is the overall well-off level, but there is still a certain gap to reach the comprehensive well-off level. Therefore, the government must realize the fact that adjusting the economic structure, increasing water saving, improving the reclaimed water reuse rate and water use efficiency, optimizing the water ecological environment, and improving the water resources carrying capacity are inevitable ways to effectively realize the sustainable development of social economy. In addition, this paper lacks in-depth research on the development and utilization of water resources in the upper and lower reaches of Zhuanglang River. For example, if the upstream self-produced water is large and the utilization rate is low, it is worth further study whether the upstream self-produced water resources can be converted into the downstream water resources availability and then the carrying capacity of water resources can be evaluated. In the future, we will take relevant measures and methods to discuss the content, so as to make the content of this paper more perfect. And due to the advanced nature of this report in the water resources carrying capacity of Gansu Province, there is no clear research on the carrying capacity of water resources in the whole province, which is also taken as the next research direction.

## Supplementary Information

Below is the link to the electronic supplementary material

Supplementary file1 (DOCX 46 KB)
