# Supplementary material for: Study on water resources carrying capacity in Zhuanglang River Basin
Source: Environ Monit Assess. 2022 Apr 22;194(5):390. doi: 10.1007/s10661-022-10027-6 (PMC9023428; doi:10.1007/s10661-022-10027-6)
Supplement: Supplementary file 1 — Supplementary file1 (DOCX 46 KB) [file 10661_2022_10027_MOESM1_ESM.docx]

**Supplementary Information**

Table S1 Social and economic indicators of each township in Zhuanglang River Basin

| County-level adminis-trative district | Serial  number | Towns | Drainage area（km^2^） | Population（Unit:10000） | |  | GDP  (100 millionyuan) | Industrial output value (100 million yuan) | Agricultural output value (100 million yuan) | Cultivated land area (ten thousand mu) | Irrigation area (ten thousand mu) | Animal husbandry area (ten thousand mu) | Grain production (10,000 tons) |
| --- | --- | --- | --- | --- | --- | --- | --- | --- | --- | --- | --- | --- | --- |
|  |  |  |  | Urban population | rural population | total |  |  |  |  |  |  |  |
| Tian | 1 | Zhuaxixiulong township | 453.6 | 0.064 | 0.272 | 0.336 | 0.216 | 0.376 | 0.088 | 1.24 | 1.24 | 35.04 | 0.176 |
| Zhu | 2 | Dachaigou town | 365.6 | 0.104 | 1.24 | 1.344 | 0.808 | 0.376 | 0.032 | 4.96 | 4.96 | 27.2 | 0.416 |
| county | 3 | Huazangsi town | 501.6 | 3.744 | 1.6 | 5.344 | 1.96 | 0.904 | 1.056 | 5.632 | 3.32 | 22.632 | 7.056 |
|  | Subtotal | | 1320.8 | 3.912 | 3.112 | 7.024 | 2.984 | 1.656 | 1.464 | 11.832 | 9.52 | 84.872 | 7.648 |
|  | 1 | Wushengyi town | 375.2 | 0.496 | 2.312 | 2.808 | 5.72 | 4.32 | 2.144 | 10.624 | 1.784 | 24.288 | 1.528 |
|  | 2 | Zhongbao town | 65.6 | 0.64 | 1.296 | 1.936 | 5.928 | 9.68 | 0.712 | 2.392 | 1.456 | 10.92 | 0.368 |
| Yong | 3 | Chengguan town | 53.6 | 3.568 | 0.232 | 3.8 | 13.84 | 0.96 | 0.568 | 1.248 | 0.792 | 4.36 | 0.216 |
| Deng | 4 | Liushu town | 320 | 0.056 | 2.16 | 2.216 | 4.528 | 0.688 | 1.296 | 5.76 | 2.384 | 29.992 | 0.864 |
| conuty | 5 | Datong town | 228 | 0.464 | 1.68 | 2.144 | 4.352 | 0.648 | 1.112 | 3.912 | 2.824 | 23.48 | 0.584 |
|  | 6 | Longquansi town | 204 | 0.368 | 1.464 | 1.832 | 4.352 | 0.32 | 1.16 | 4.416 | 2.592 | 39.272 | 0.864 |
|  | 7 | Hongcheng town | 270.4 | 0.648 | 1.488 | 2.136 | 4.176 | 0.88 | 0.68 | 4.312 | 1.776 | 28.296 | 0.8 |
|  | 8 | Kushui town | 352 | 0.344 | 2.256 | 2.6 | 4.624 | 2.56 | 0.944 | 2.992 | 1.928 | 37.52 | 0.504 |
|  | Subtotal | | 1868.8 | 6.584 | 12.888 | 19.472 | 47.52 | 20.056 | 8.616 | 35.656 | 15.536 | 198.128 | 5.728 |
| Xigu District | 1 | Hekou town | 16 | 0.016 | 0.144 | 0.16 | 0.016 | 0 | 0 | 0.128 | 0 | 0 | 0.016 |
|  | Subtotal | | 16 | 0.016 | 0.144 | 0.16 | 0.016 | 0 | 0 | 0.128 | 0 | 0 | 0.016 |
| Total |  |  | 3205.6 | 10.512 | 16.144 | 26.656 | 54.56 | 21.712 | 10.08 | 47.616 | 25.056 | 283 | 13.392 |

Data source: compiled by the author

Table S2 Characteristic values of township water resources in Zhuanglang River Basin

| County-level administrative region | Serial number | | Townships | The basin area（km^2^） | Surface water resources (${10}^{4}$m³） |
| --- | --- | --- | --- | --- | --- |
|  |  |  |  |  |  |
| Tianzhu County | 1 | | Zhuaxixiulong township | 567 | 5387 |
|  | 2 | | Dachaigou town | 457 | 3610 |
|  | 3 | | Huazangsi town | 627 | 3919 |
|  | Subtotal | | | 1651 | 12916 |
| Yongdeng County | 1 | | Wushengyi town | 469 | 3142 |
|  | 2 | | Zhongbao town | 82 | 369 |
|  | 3 | | Chengguan town | 67 | 184 |
|  | 4 | | Liushu town | 400 | 1900 |
|  | 5 | | Datong town | 285 | 1069 |
|  | 6 | | Longquansi town | 255 | 650 |
|  | 7 | | Hongcheng town | 338 | 1200 |
|  | 8 | | Kushuui town | 440 | 999 |
|  | Subtotal | | | 2336 | 9513 |
| Xigu County | 1 | Hekou town | | 20 | 3.4 |
|  | Subtotal | | | 20 | 3.4 |
| Total | | | | 4007 | 22432 |

Data source: compiled by the author

Table S3 Characteristic values of water resources in towns and townships of Zhuanglanhe River Basin

| County-level administrative district | Serial number | Townships | Drainage area | Surface water resources |
| --- | --- | --- | --- | --- |
|  |  |  | （km^2^） | (10000 m³） |
|  | 1 | Zhuaxixiulong township | 453.6 | 4309.6 |
| Tianzhu county | 2 | Dachaigou town | 365.6 | 2888 |
|  | 3 | Huazangsi town | 501.6 | 3135.2 |
|  | Subtotal |  | 1320.8 | 10332.8 |
|  | 1 | Wushengyi town | 375.2 | 2513.6 |
|  | 2 | Zhongbao town | 65.6 | 295.2 |
|  | 3 | Chengguan town | 53.6 | 147.2 |
| Yongdeng county | 4 | Liushu town | 320 | 1520 |
|  | 5 | Datong town | 228 | 855.2 |
|  | 6 | Longquansi town | 204 | 520 |
|  | 7 | Hongcheng town | 270.4 | 960 |
|  | 8 | Kushui town | 352 | 799.2 |
|  | Subtotal |  | 1868.8 | 7610.4 |
| Xigu District | 1 | Hekou town | 16 | 2.72 |
|  | Subtotal |  | 16 | 2.72 |
| Total |  |  | 3205.6 | 17945.6 |

Data source: compiled by the author

Table S4 Availability of surface water in each township of Zhuanglang River Basin

| County-level administrative district | Serial number | Townships | Drainage area（km^2^） | Surface water resources | Available surface water | |
| --- | --- | --- | --- | --- | --- | --- |
|  |  |  |  | (10000m³） | (10000m³） |  |
|  | 1 | Zhuaxixiulong township | 453.6 | 4309.2 | 2301.12 |  |
| Tianzhu county | 2 | Dachaigou town | 365.6 | 2888.24 | 1542.32 |  |
|  | 3 | Huazangsi town | 501.6 | 3135.04 | 1674.08 |  |
|  | Subtotal |  | 1320.8 | 10332.48 | 5517.52 |  |
|  | 1 | Wushengyi town | 375.2 | 2513.84 | 1123.68 |  |
|  | 2 | Zhongbao town | 65.6 | 295.2 | 131.92 |  |
|  | 3 | Chengguan town | 53.6 | 147.4 | 65.92 |  |
| Yongdeng county | 4 | Liushu town | 320 | 1520 | 679.44 |  |
|  | 5 | Datong town | 228 | 855 | 382.16 |  |
|  | 6 | Longquansi town | 204 | 520.2 | 232.56 |  |
|  | 7 | Hongcheng town | 270.4 | 959.92 | 429.12 |  |
|  | 8 | Kushuui town | 352 | 799.04 | 357.2 |  |
|  | Subtotal |  | 1868.8 | 7610.6 | 3402 |  |
| Xigu District | 1 | Hekou town | 16 | 2.72 | 1.2 |  |
|  | Subtotal |  | 16 | 2.72 | 1.2 |  |
| Total |  |  | 3205.6 | 17945.8 | 8920.72 |  |

Data source: compiled by the author

Table S5 Distribution table of groundwater resources in towns and townships of Zhuanglanhe River Basin

| County-level administrative district | Serial number | Townships | Drainage area | Groundwater resources |
| --- | --- | --- | --- | --- |
|  |  |  | （km^2^） | (10000m³) |
| Tianzhu County | 1 | Zhuaxixiulong township | 453.6 | 4308.8 |
|  | 2 | Dachaigou town | 365.6 | 2888 |
|  | 3 | Huazangsi town | 501.6 | 3135.2 |
|  | Subtotal |  | 1320.8 | 6772 |
| Yongdeng County | 1 | Wushengyi town | 375.2 | 495.2 |
|  | 2 | Zhongbao town | 65.6 | 86.4 |
|  | 3 | Chengguan town | 53.6 | 70.4 |
|  | 4 | Liushu town | 320 | 422.4 |
|  | 5 | Datong town | 228 | 300.8 |
|  | 6 | Longquansi town | 204 | 269.6 |
|  | 7 | Hongcheng town | 270.4 | 356.8 |
|  | 8 | Kushuui town | 352 | 464.8 |
|  | Subtotal |  | 1868.8 | 2468 |
| Xigu District | 1 | Hekou town | 16 | 64.8 |
|  | Subtotal |  | 16 | 64.8 |
| Total |  |  | 3205.6 | 9304.8 |

Data source: compiled by the author

Table S6 Groundwater recoverable amount of each township in Zhuanglang River Basin

| County-level administrative district | Serial number | Townships | | Drainage area（km^2^） | Groundwater resources (10000m³) | Available groundwater  (10000m³) |
| --- | --- | --- | --- | --- | --- | --- |
|  |  |  |  |  |  |  |
| Tianzhu County | 1 | Zhuaxixiulong township | | 453.6 | 4308.8 | 1378.816 |
|  | 2 | Dachaigou town | | 365.6 | 2888 | 924.16 |
|  | 3 | Huazangsi town | | 501.6 | 3135.2 | 1003.264 |
|  | Subtotal | | | 1320.8 | 6772 | 3306.24 |
| Yongdeng County | 1 | Wushengyi town | | 375.2 | 495.2 | 123.8 |
|  | 2 | Zhongbao town | | 65.6 | 86.4 | 21.6 |
|  | 3 | Chengguan town | | 53.6 | 70.4 | 17.6 |
|  | 4 | Liushu town | | 320 | 422.4 | 105.6 |
|  | 5 | Datong town | | 228 | 300.8 | 75.2 |
|  | 6 | Longquansi town | | 204 | 269.6 | 67.4 |
|  | 7 | Hongcheng town | | 270.4 | 356.8 | 89.2 |
|  | 8 | Kushuui town | | 352 | 464.8 | 116.2 |
|  | Subtotal | | | 1868.8 | 2468 | 616.6 |
| Xigu District | 1 | | Hekou town | 16 | 64.8 | 16.2 |
|  | Subtotal | | | 16 | 64.8 | 16.2 |
| Total | | | | 3205.6 | 9304.8 | 3939.04 |

Data source: compiled by the author

Table S7 Relationship between mood operator and relative membership degree of quantitative scale

| Mood operator | Quantitative scale | | Relative membership degree | |
| --- | --- | --- | --- | --- |
| samely | 0.50 | 0.525 | 1.000 | 0.905 |
| little | 0.55 | 0.575 | 0.818 | 0.739 |
| slightly | 0.60 | 0.625 | 0.667 | 0.600 |
| relatively | 0.65 | 0.675 | 0.538 | 0.481 |
| clearly | 0.70 | 0.725 | 0.429 | 0.379 |
| markedly | 0.75 | 0.775 | 0.333 | 0.290 |
| very | 0.80 | 0.825 | 0.250 | 0.212 |
| bitterly | 0.85 | 0.875 | 0.176 | 0.143 |
| exceedingly | 0.90 | 0.925 | 0.111 | 0.081 |
| extremely | 0.95 | 0.975 | 0.053 | 0.026 |
| Incomparably | 1.00 | - | 0 | - |

Data source: compiled by the author

Table S8 Evaluation index values of each township in Zhuanglang River Basin

| Comprehensive evaluation index | Water resources system | | | Social system | | | | | Economic system | | | |
| --- | --- | --- | --- | --- | --- | --- | --- | --- | --- | --- | --- | --- |
|  | Water resource modulus | Development and utilization rate of water resources | Water supply modulus | Population density | Urbanization rate | Population growth rate | Urban domestic water quota | Rural domestic water quota | Per capita GDP | Industrial water consumption per 10000 yuan of added value | Irrigation rate of plough | grain yield per hectare |
| Index positive and negative | positive | negative | positive | negative | positive | negative | positive | positive | negative | negative | positive | positive |
| Zhuaxixiulong township | 8.68184 | 0.09872 | 1.07136 | 5.92592 | 0.1524 | 0.03528 | 29.6 | 13.6 | 0.50552 | 21.448 | 0.8 | 0.11352 |
| Dachaigou town | 7.155184 | 0.17288 | 1.546008 | 29.40912 | 0.06192 | 0.05496 | 26.56 | 14.4 | 0.48216 | 128.688 | 0.8 | 0.06744 |
| Huazangsi town | 6.82348 | 0.19456 | 1.659662 | 79.7448 | 0.53504 | 0.05216 | 31.2 | 16.8 | 0.31416 | 72.408 | 0.4716 | 1.00224 |
| Wushengyi town | 5.90584 | 0.31832 | 2.350115 | 59.872 | 0.14128 | 0.04208 | 51.76 | 23.2 | 1.6296 | 22.464 | 0.13432 | 0.11504 |
| Zhongbao town | 2.985368 | 4.8852 | 18.2301 | 236.0968 | 0.26448 | 0.06248 | 76.72 | 39.2 | 2.44952 | 25.632 | 0.48696 | 0.12304 |
| Chengguan town | 1.917608 | 3.75888 | 9.010048 | 567.164 | 0.75112 | 0.07384 | 88 | 40 | 2.91368 | 38.592 | 0.50768 | 0.1384 |
| Liushu town | 2.76 | 0.91728 | 3.164571 | 55.48 | 0.02136 | 0.06768 | 69.92 | 32 | 1.63224 | 28.512 | 0.33112 | 0.12 |
| Datong town | 2.6414 | 1.60584 | 5.302054 | 75.228 | 0.17312 | 0.0612 | 71.2 | 32 | 1.62384 | 45.504 | 0.57752 | 0.11936 |
| Longquansi town | 2.10196 | 1.9452 | 5.110873 | 71.84312 | 0.16072 | 0.04608 | 75.04 | 36 | 1.9004 | 22.176 | 0.4696 | 0.15648 |
| Hongcheng town | 2.680712 | 0.85192 | 2.854727 | 63.1952 | 0.24272 | 0.06344 | 71.6 | 32 | 1.564 | 19.872 | 0.32952 | 0.1484 |
| Kushuui town | 1.549088 | 1.22256 | 2.367346 | 59.09088 | 0.10584 | 0.04352 | 56.72 | 28 | 1.42272 | 7.488 | 0.51552 | 0.13472 |
| Hekou town | 0.936 | 0.98624 | 1.153858 | 80 | 0.08 | 0.06496 | 74 | 40 | 0.06 | 0 | 0 | 0.1 |

Data source: compiled by the author

| Comprehensive evaluation index | Economic system | | | ecosystem |  | Comprehensive coordination | | |
| --- | --- | --- | --- | --- | --- | --- | --- | --- |
|  | Reuse rate of industrial water | Water consumption per unit grain | Irrigation water quota | Forest and animal husbandry coverage | Groundwater extraction rate | Water resources per capita | Water consumption per unit GDP | Ecological water use rate |
| Index positive and negative | positive | negative | negative | positive | negative | positive | positive | positive |
| Zhuaxixiulong township | 0.2896 | 2563.632 | 0.02911 | 0.412 | 0 | 11720.57 | 2288.852 | 0.016 |
| Dachaigou town | 0.2888 | 954.488 | 0.006441 | 0.3968 | 0.0048 | 1946.381 | 697.7344 | 0.03104 |
| Huazangsi town | 0.2968 | 81.1784 | 0.013802 | 0.24064 | 0.0032 | 684.5312 | 423.9248 | 0.03136 |
| Wushengyi town | 0.288 | 363.9784 | 0.02494 | 0.34528 | 0.2512 | 789.1282 | 154.1544 | 0.016 |
| Zhongbao town | 0.2888 | 1233.04 | 0.024932 | 0.88784 | >100% | 101.157 | 201.7367 | 0.008 |
| Chengguan town | 0.288 | 1188.144 | 0.025923 | 0.43384 | >100% | 27.04842 | 34.89442 | 0.016 |
| Liushu town | 0.2968 | 1037.776 | 0.030089 | 0.49984 | 68.56 | 397.9812 | 223.6446 | 0.016 |
| Datong town | 0.2976 | 1819.176 | 0.030096 | 0.5492 | >100% | 280.8955 | 277.7731 | 0.016 |
| Longquansi town | 0.288 | 1128.888 | 0.030104 | 1.02672 | 0.7824 | 234.0611 | 239.5722 | 0.008 |
| Hongcheng town | 0.2904 | 834.4 | 0.030068 | 0.55808 | 0.2608 | 339.3558 | 184.8464 | 0.016 |
| Kushuui town | 0.288 | 1434.92 | 0.030008 | 0.56848 | 0 | 209.723 | 180.2133 | 0.016 |
| Hekou town | 0 | 840 | 0 | 0 | 0 | 93.6 | 1538.478 | 0.07336 |

Data source: compiled by the author

Table S9 Classification systems and classification standard of water resources carrying capacity

| Basic types | Subtype | Relative carrying capacity index RCI |
| --- | --- | --- |
|  | strong（ⅠA） | RCI≥2 |
| overload（Ⅰ） | medium（ⅠB） | 1.5≤RCI＜2 |
|  | weak（ⅠC） | 1＜RCI＜1.5 |
| crisis（Ⅱ） |  | RCI=1 |
|  | weak（ⅢC） | 2/3＜RCI＜1 |
| unload（Ⅲ） | medium（ⅢB） | 0.5＜RCI＜2/3 |
|  | strong（ⅢA） | RCI＜0.5 |

Data source: compiled by the author
